# Study protocol for a double-blinded randomized clinical trial evaluating effectiveness of neurodynamic approach in lower limb for diabetic peripheral sensory neuropathy

**DOI:** 10.1371/journal.pone.0348347

**Published:** 2026-05-12

**Authors:** Ambika Kormoker, K M Amran Hossain, Md. Zahirul Islam, Abdullah Al Gaddafi, Kazi Md Azman Hossain, Md. Feroz Kabir, Md. Zahid Hossain, Ehsanur Rahman, Kabir Hossain, Abid Hasan Khan

**Affiliations:** 1 Department of Physiotherapy and Rehabilitation, Jashore University of Science and Technology (JUST), Jashore, Bangladesh; 2 Department of Orthopaedic Surgery, Jashore Medical College, Jashore, Bangladesh; 3 Department of Diabetic Foot Surgery, Ibn Sina Diagnostic & Hospitals, Jashore, Bangladesh; Rutgers University Newark, UNITED STATES OF AMERICA

## Abstract

**Background:**

Diabetic peripheral sensory neuropathy (DPSN) presents with pain or neuropathy symptoms and disturbances in lower limb sensory functions, leading to complicated diabetic foot conditions and declining quality of life. There is a research gap on the non-pharmacological conservative approach to this disabling condition. The objective of this study is to evaluate the effectiveness of the neurodynamic approach, along with electrophysical stimulation, in reducing the severity of neuropathic symptoms and improving the quality of life in patients with DPSN.

**Methods:**

An assessor and patient-blinded randomized clinical trial (RCT) on 90 DPSN cases through hospital-based simple random sampling. Participants will be randomly assigned equally to either the Neurodynamic approach or the Sham neurodynamic approach group by concealed allocation between April and August 2025 at the Nurul Islam Diabetic Center in Jashore, Bangladesh. The experimental group will receive the Neurodynamic approach, and the control group will receive the sham approach; however, both groups will receive transcutaneous electrical nerve stimulation (TENS) and home exercises for 12 sessions over 4 weeks. A pretest will be taken at baseline, a posttest after 4 weeks, and a follow-up evaluation after 12 weeks. In addition to outcome measures, manageable and fatal adverse events will be documented and reported.

**Discussion:**

This trial will be implemented in the non-pharmacological management of prevalent cases with DPSN and diabetic foot, sparing the burden of polypharmacy in people living with diabetes. Moreover, a positive result may guide clinicians toward an alternative in evidence-based practice, especially in a low-resource country like Bangladesh. This single-center trial may be limited by a lack of external generalizability, but it will lead to multicenter, large-sample studies in the future.

**Trial registration:**

Registered from Clinical Trial Registry of India CTRI/2025/01/079801 [Registered on: 30/01/2025] Trial Registered Prospectively.

## Introduction

Diabetes mellitus (DM) is a chronic condition resulting from insufficient insulin in the body or reduced insulin effectiveness, leading to high blood sugar levels [1]. Diabetes is classified into two main types: type-1 diabetes, where the body does not produce insulin and requires external insulin for survival, and type-2 diabetes, where the body becomes resistant to insulin. Type-2 diabetes is the most common, affecting around 90–94% of people with diabetes worldwide [2]. The prevalence of diabetes continues to rise, with an estimated 422 million people affected worldwide, especially in low- and middle-income countries [3]. One of the most serious complications of diabetes is diabetic peripheral neuropathy (DPN), a condition that occurs when high blood sugar damages the nerves, particularly in the legs and feet [4]. Nearly 50% of people with diabetes develop DPN, making it one of the leading causes of chronic pain, numbness, and mobility problems. Symptoms of DPN include tingling, burning pain, numbness, and reduced sensation, especially in the lower limbs [5]. Diabetic neuropathy is usually classified into two main categories: autonomic neuropathy and sensory neuropathy. The condition (DPN) presents as sensory, motor, and autonomic dysfunction, leading to symptoms such as pain, weakness, tingling, and numbness [6]. Diabetic peripheral sensory neuropathy (DPSN) is characterized by distal symmetric neuropathy that involves numbness, discomfort, loss of sensation, and abnormal sensation [7]. Affected individuals of DPSN often experience altered sensations, including electric shock-like pain, reduced heat and touch sensitivity, and progressive loss of protective sensation [8]. These issues not only affect daily life but also increase the risk of serious complications like foot ulcers, infections, and amputations. Remarkably, DPSN is responsible for 80% of diabetes-related foot ulcers, which can lead to severe infections and a high risk of death [9,10].

DPSN is a common problem in both type-1 and type-2 diabetes, but research shows that it develops differently in each condition. The differences in metabolism between T1DM and T2DM lead to different types of nerve damage [11]. It develops from a combination of metabolic and blood vessel-related problems that impair nerve function [12]. Prolonged high blood sugar levels lead to oxidative stress, inflammation, and reduced blood supply, which harm nerve fibers [13]. Over time, these changes cause loss of protective nerve coverings (demyelination) and nerve degeneration, leading to sensory and motor dysfunction [14]. One of the major biomechanical effects of DPSN is reduced nerve mobility and flexibility, which affects nerve gliding through tissues [15]. Research has shown that people with DPSN exhibit reduced nerve mobility and stiffness in the lower limbs, particularly during movements such as ankle dorsiflexion and hip flexion [16]. This reduced nerve flexibility contributes to increased pain, muscle weakness, and difficulty with movement, making walking and daily activities challenging [17]. As a result, individuals with DPSN are at a higher risk of falls, injuries, and disability [18]. The biomechanical changes resulting from DPSN may translate to increased plantar pressures in the foot, which contribute to the pathogenesis and development of foot ulcers, especially in the forefoot [19]. In particular, the first metatarsophalangeal joint has been implicated as a site of biomechanical dysfunction leading to elevated plantar pressures during gait, promoting ulceration at this site [20,21].

Both pharmaceutical (medications) and non-pharmacological (non-medication) treatments are used to manage DPSN [22]. Antidepressants, anticonvulsants, and gabapentinoids are examples of painkillers that can help decrease symptoms, though they do not totally treat DSPN and frequently have negative side effects [23]. In recent years, physiotherapy-based treatments have gained attention as an alternative approach to managing DPSN. Neurodynamic mobilization is one of these that has to improve nerve function and reduce pain [24,25]. Specific nerve movements are used in neurodynamic mobilization to increase blood flow to the affected areas, decrease stiffness, and improve the nerve’s capacity to glide smoothly [26]. According to studies, neurodynamic exercises can help people with mild-to-severe DPSN move more freely, reduce pain, and improve lower-limb flexibility [27].

There is still limited research on the effectiveness of neurodynamic mobilization for DPSN in Bangladesh. Most studies have been conducted in animals or have focused on a single nerve, making it difficult to draw strong conclusions for human patients [28]. This study aims to fill this research gap by evaluating the effects of neurodynamic mobilization combined with foot exercises on neuropathy severity, pain, sensory disturbances, and quality of life in people with DPSN. The study is based on the hypothesis that neurodynamic mobilization will significantly improve nerve function, reduce pain, and enhance the quality of life (QoL) compared to standard treatments. The objectives of the study are (1) to find out the outcome of the neurodynamic approach on the severity of neuropathy, (2) to elicit the effectiveness of the neurodynamic approach in reducing neuropathic pain, and (3) to evaluate the impact of the neurodynamic approach on quality of life in individuals with lower limb DPSN.

## Methods

### Study design

**Fig 1:**
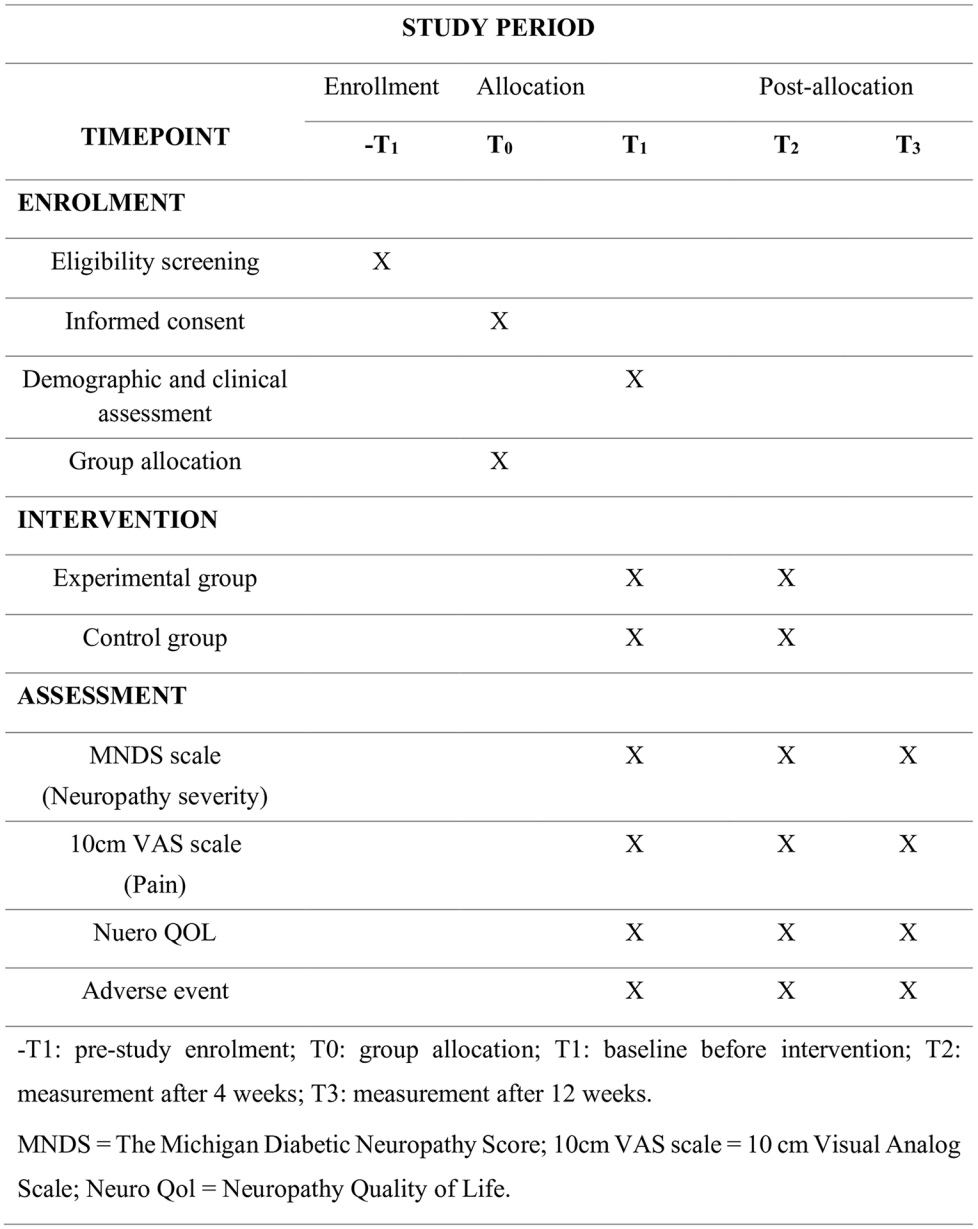
SPIRIT schedule of enrolment, interventions, and assessments.

**Fig 2:**
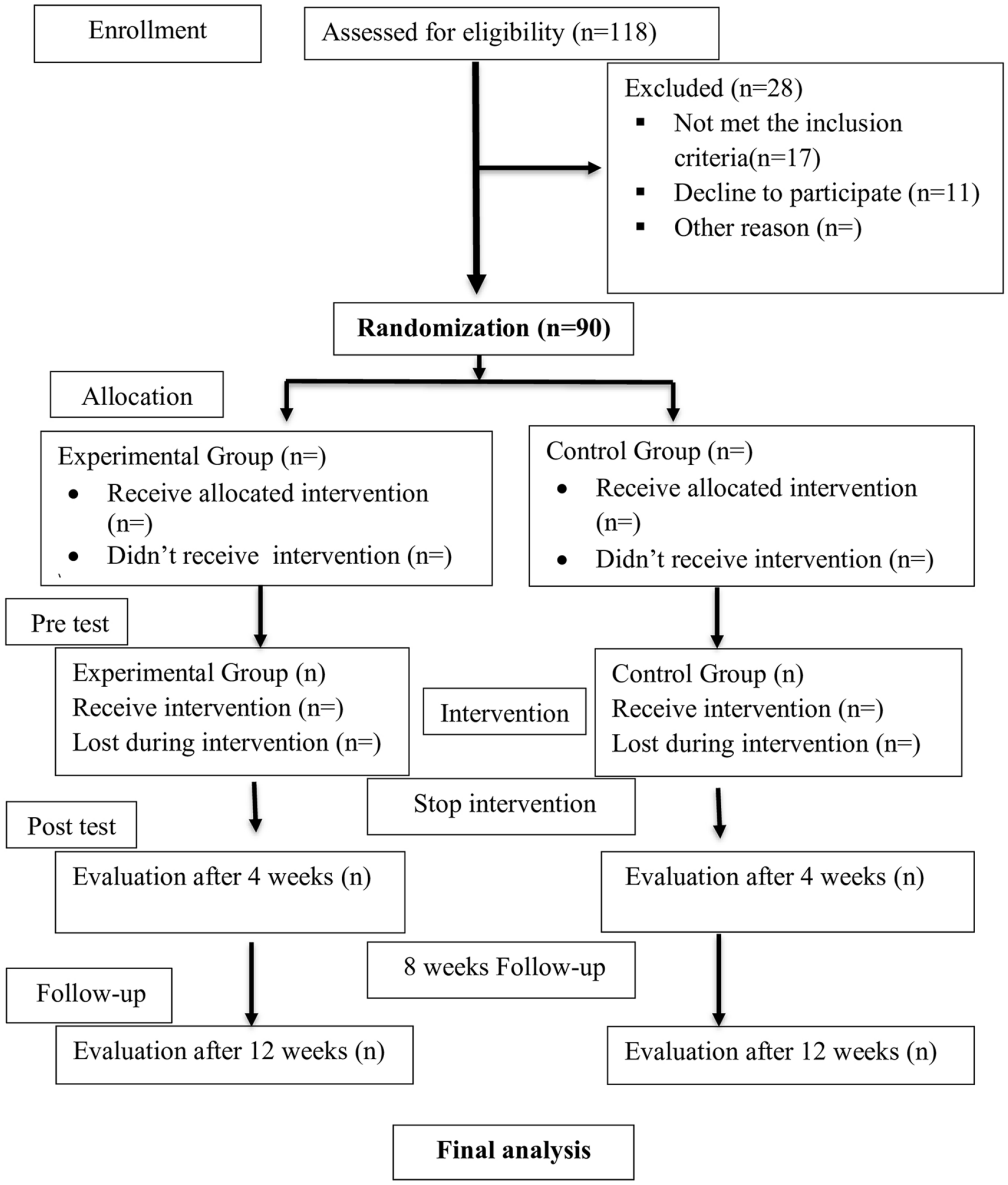
CONSORT flow diagram of participant progression.

The study will be an assessor and patient-blinded Randomized Clinical Trial (RCT) at the Nurul Islam Diabetic Center in Jashore, Bangladesh. Ninety (90) patients diagnosed with DPSN by a diabetic foot surgeon and experiencing pain, burning, or tingling in the lower limbs or peripheral nerves will be randomly recruited via hospital-based simple randomization between April 2025 and August 2025. The recruited patients will be randomly allocated to either the Neurodynamic Mobilization (Experimental) (n = 45) or Sham Neurodynamic Mobilization (Control) (n = 45) groups in a 1:1 ratio using simple random sampling. The experimental group will receive Neurodynamic mobilization, and the control group will receive Sham Neurodynamic Mobilization for 12 sessions in 4 weeks; both groups will receive common foot exercises and transcutaneous electrical nerve stimulation (TENS) as a common treatment. The post-test will be taken 4 weeks after baseline assessment and recruitment, and the follow-up will be taken 12 weeks after the post-test. The follow-up assessment of the last recruited participant will end by August 2025. The study protocol will follow the Fig 1. SPIRIT (Standard Protocol Items: Recommendations for Interventional Trials) guideline of the study protocol, ensuring a clear and comprehensive study design, including participant recruitment, randomization, intervention allocation, and follow-up assessments.. The results will be reported in accordance with the CONSORT (Consolidated Standards of Reporting Trials) flow diagram (Fig 2) of participant progression, which shows the flow of participants through the enrollment, allocation, follow-up, and analysis phases of the randomized clinical trial.

### Sample size calculation

The number of patients was determined using G*Power (version 3.1.9.7) based on changes in neuropathic pain scores from a study by [29], which examined the effect of the Michigan Diabetic Neuropathy Score (MDNS), a validated tool used to assess the severity of diabetic peripheral neuropathy. 42 subjects per group were shown to be necessary based on an effect size of 0.62, an alpha level of 0.05, and a power of 0.8. Hence, the total sample size was 84. [30]. We anticipate a 10% dropout rate, so we will recruit 90 participants in total and allocate them equally between the two groups.

### Eligibility criteria

The study's inclusion and exclusion criteria will be applied to participants through a screening process conducted by a diabetic foot surgeon and a consultant physiotherapist.

### Inclusion

1. Adults between 40 and 70 years old [24].
2. Who have been diagnosed with Type-2 DPSN & Type-1 DPSN [31]
3. Individuals who will give informed consent.
4. The Michigan Diabetic Neuropathy Score (MDNS) is more than 6 [32].
5. Symmetrical or asymmetric peripheral neuropathy accompanied by lower extremities discomfort lasting more than six months [32].

### Exclusion

1. Last six months, sciatica and radiculopathy of low back pain (lasting at least three days) [33].
2. Musculoskeletal dysfunctions such as lumbar disc prolapses, scoliosis, and lumbar spine-related radiculopathy [34].
3. Chemical, drug, or alcohol dependency.
4. Medical diagnosis of a herniated disk in the lumbar spine [35].
5. Pregnancy
6. Surgery for back pain or any other type of surgery
7. Amputation of lower limb [36].

### Intervention

Participants will be randomly allocated to either the experimental group (neurodynamic mobilization) or the control group (sham neurodynamic mobilization). Both groups will additionally receive standard care consisting of transcutaneous electrical nerve stimulation (TENS) and a structured foot exercise program. All interventions will be delivered by trained physiotherapists following a standardized protocol to ensure consistency.

Experimental group (Neurodynamic Mobilization)

Participants in the experimental group will receive neurodynamic mobilization targeting the sciatic, tibial, sural, and common peroneal nerves.

General Treatment ParametersRepetitions:10 repetitions per set

Sets: 2–3 sets per session,

Hold time: 6–15 seconds per repetition.

Session duration: Approximately 30 minutes

Frequency: 3 sessions per week for 4 weeks.

Progression: The intensity and range of motion will be gradually increased based on the patient's tolerance and response to symptoms.

#### Sciatic nerve mobilization.

Position: The patient lies supine with pillows and maintains the therapist's proper position.

Technique: The ankle is positioned in dorsiflexion, and the therapist passively lifts the patient’s straight leg while keeping the knee fully extended (to increase tension on the nerve), and continues raising the leg until the patient feels tension or symptoms (e.g., pain, tingling). Once symptoms (such as pain or discomfort) appear, the therapist will lower the hip slightly to a pain-free position. Then, oscillatory mobilizations will be applied. The hip will be alternately moved between hip flexion (nerve loading) with ankle dorsiflexion (nerve loading) and hip extension (nerve unloading) with ankle plantarflexion (nerve unloading) [37–39].

#### Tibial nerve mobilization.

Position: The patient is in the supine position with pillows, and the therapist maintains proper position.

Technique: The therapist will place the ankle in dorsiflexion and eversion while the knee remains straight. The therapist then slowly moves the hip into flexion (lifting the leg) and the knee into extension to allow the nerve to glide smoothly. Once symptoms appear, the therapist will lower the hip slightly to a pain-free position. The hip will then be alternately flexed (nerve unloading) with ankle dorsiflexion and eversion (nerve loading) and extended (nerve loading) with ankle plantarflexion (nerve unloading) [37–39].

#### Common peroneal nerve mobilization.

Position: The patient lies supine with pillows and maintains the therapist's proper position.

Technique: The therapist will apply ankle plantarflexion and inversion, then passively flex the hip while keeping the knee extended. Once symptoms appear, the therapist will adjust the hip and knee to a pain-free position. The hip will be moved alternately in flexion of the hip joint (nerve unloading) with ankle plantarflexion and inversion (nerve loading) and then into extension (nerve loading) with a neutral ankle position (nerve unloading) [37–39].

#### Sural nerve mobilization.

Position: The patient lies supine with pillows and maintains the therapist's proper position.

Technique: The therapist will place the ankle and dorsiflexion and invert the ankle while passively flexing the hip, keeping the knee extended. Once symptoms appear, the therapist will adjust the hip and knee to a pain-free position, then alternately move into hip flexion (nerve unloading) with ankle dorsiflexion and inversion (nerve loading), and then into hip extension (nerve loading) with ankle dorsiflexion (nerve unloading) [37–39].

### Control group (Sham Neurodynamic Mobilization)

Participants in the control group will receive sham neurodynamic mobilization, in which similar movements are performed without applying nerve-tension techniques.

Position: The patient lies supine with pillows and maintains the therapist's proper position.

Technique: The therapist will place the patient's ankle in dorsiflexion and move the hip into abduction and flexion instead of true sciatic nerve-loading movements**.** The knee remains extended, and the therapist stops at a pain-free position**.** Each technique will be performed for 10 repetitions, with a hold time of a few seconds per repetition.

#### Additional interventions for both groups.

***Foot Exercises:*** Both groups will perform foot exercises to improve mobility and sensory function. Exercises include: Foot tapping,

“V” shape making (foot movements creating a “V” shape),

Ankle rotations,

Tennis ball rolling (rolling a ball under the foot),

Foot somatosensory exercise (rolling a small ball under the foot for sensory stimulation)

Frequency:3–6 times per session for both feet**.** [40].

***Transcutaneous Electrical Nerve Stimulation (TENS):*** Both groups will receive TENS therapy

**Frequency:** 3 sessions per week for 4 weeks;

**Duration:** 20 minutes per session;

**Settings:** 80 Hz frequency, 150 µs pulse width

[41].

#### TENS electrode placement.

The placement of TENS electrodes depends on the target nerve being treated. Below are the recommended electrode placements for each nerve-

**Sciatic Nerve-** One electrode over the sciatic notch or gluteal area, near the piriformis muscle. Position the second electrode on the middle back of the thigh [42].

**Tibial Nerve-** One electrode just above the medial ankle (posterior to the medial malleolus). The second electrode on the calf, midway between the knee and ankle (posterior aspect) [43].

**Common Peroneal Nerve-** One electrode over the head of the fibula (just below the outer knee). And the second electrode along the lateral shin, midway between the knee and ankle [44].

**Sural Nerve-** One electrode over the mid-calf (posterior aspect, near the gastrocnemius muscle), the second electrode just above the lateral ankle (posterior to the lateral malleolus) [45–47].

#### Therapist training and standardization.

All interventions will be delivered by trained physiotherapists following a standardized protocol to ensure consistency across sessions.

### Outcome measures

**Socio-demographic Information:** The questionnaire will include socio-demographics such as patient ID, age, address, gender, occupation, education level, marital status, height, weight, BMI, etc.

**Primary outcomes**

1. The Michigan Diabetic Neuropathy Score (MDNS): It is a validated scoring system for the severity of diabetic peripheral neuropathy (DPN), which is measured by MDNS.

It comprises three sensory tests, totaling 46 points per limb. The Vibration Perception Threshold (VPT) is assessed using a 128 Hz tuning fork placed on the bony part of the big toe's joint to measure nerve response to vibrations. Light touch sensation is tested by applying a 10-gram monofilament on the top of the big toe, between the nail fold and joint. Pinprick sensation is evaluated using a standard pin on the top of the foot to check the response to sharp touch. These tests help assess the degree of nerve damage in individuals with DPN [48–50].

### Secondary outcomes

**1. 10 cm Visual Analog Scale (VAS):**
  The Visual Analog Scale (VAS) is a widely used tool for measuring pain and is known for its high accuracy (ICC = 0.98). Participants will be asked to place a mark on a straight line to show how much pain they are feeling at that moment. The pain level is then measured in millimeters to quantify pain level [51–54].
**2. Neuropathy-specific quality of life questionnaire (Neuro QoL):**
  The Neuro-QoL questionnaire is used to assess and monitor patients with diabetic peripheral neuropathy (DPN) both during and after treatment. It helps evaluate how DPN affects daily life, both physically and mentally**,** as well as overall quality of life**.** The questionnaire consists of 27 questions divided into six categories**,** each rated on a 5-point scale (1 = never, 5 = all the time) based on how often symptoms occur. The six categories include pain and tingling (paresthesia), changes in foot sensation and temperature, difficulty with balance while standing and walking, limitations in daily activities, emotional and physical dependence in relationships, and emotional distress**.** Additionally, two extra questions assess the overall impact of neuropathy on life [51,53].

### Ethical issues and informed consent

This study has been approved by the ethical committee of the Department of Physiotherapy and Rehabilitation at Jashore University of Science and Technology, Bangladesh (Approval No.: PTR-JUST/IRB/2025/01/222564). The Clinical Trials Registry-India has registered it (CTRI/2025/01/079801), and it will adhere to the Declaration of Helsinki and other international ethical standards. Participants will be given comprehensive information on the study's goals, methods, potential risks, and advantages prior to enrollment. They will provide written consent in either English or Bangla to ensure they fully understand. At any point, participants are free to withdraw from the research without facing any adverse effects.

### Study procedure

A thorough physical examination of each patient will be conducted by a diabetic foot surgeon and a consultant physiotherapist following the collection of demographic information and medical screening results. After recruitment, participants will be briefed on the participant information sheet, informed consent will be obtained, and they will be sent to a blinded assessor for baseline socio-demographic and clinical examination. After completing the baseline, the participants will be sent to a separate room to receive the intervention in a concealed envelope. After completing interventions, participants will be appointed to the assessor for outcome evaluation and briefed for the follow-up evaluations. During follow-up time, participants will be contacted over the phone, and an appointment will be scheduled with the blinded assessor for clinical evaluations. Participants will receive the treatment free of charge, and they will cover only the transport costs.

### Randomization procedures

DPSN patients would be selected according to the inclusion and exclusion criteria from the Nurul Islam Diabetic Center in Jashore, Bangladesh. There will be patient enrollment, and after baseline assessment, participants will be randomly allocated to either the intervention or control group using a computer-generated randomization sequence created by an independent researcher not involved in recruitment, assessment, or treatment. A simple random sampling method will be used to ensure equal distribution between groups. The investigator, who will not participate in the evaluation or interventions, will assign participants. Participants will receive all information that relates to the goals of the investigation and the approach used. Written consent in Bangla and/or English will be given to each patient. As research participants, they will be informed of their rights, and all of their personal data will be kept private.

### Blinding

This trial will be participant- and assessor-blinded. Participants will not be informed of their group allocation, as both interventions are structurally similar, thereby reducing the likelihood of identification. Outcome assessors will remain blinded to minimize assessment bias. Physiotherapists administering the treatments cannot be blinded due to the nature of the interventions but will have no role in outcome evaluation, data handling, or analysis. Allocation concealment will be ensured via a secure randomization system managed by an independent researcher uninvolved in recruitment, treatment, or assessment. To maintain blinding, participants will be instructed not to disclose their intervention during follow-up, and the effectiveness of blinding may be evaluated at study completion by querying participants and assessors about perceived group assignments.

### Data audit and management

Data will undergo two checks to ensure accuracy and will be frequently verified by an external reviewer from the Department of Mathematics at Jashore University of Science & Technology. Data will be audited in a printed questionnaire, and in Excel formats to examine redundancy, outliers, and de-identified for the data analysis process.

### Data analysis

All statistical analyses will be performed using appropriate statistical software (SPSS Statistics, version 23). Descriptive statistics will be used to summarize baseline characteristics, with continuous variables presented as mean ± standard deviation (SD) and categorical variables as frequencies and percentages. A p-value < 0.05 will be considered statistically significant, with a 95% confidence interval (CI). The Shapiro-Wilk test and graphical analysis will be used to assess whether the data are normally distributed. A repeated-measure ANOVA will be used to analyze group differences (intervention vs. control), changes over time, and the group-by-time interaction to assess treatment effects. Appropriate nonparametric tests, such as the Friedman test for within-group comparisons and the Mann–Whitney U test for between-group comparisons, will be employed if the assumptions required for parametric testing are not met. When appropriate, post hoc analyses will be carried out, and Type I error will be controlled using the Bonferroni correction. All randomized participants will be included in the analysis using an intention-to-treat (ITT) strategy, and missing data will be handled appropriately based on the last observation carried forward method.

### Trial monitoring

The chairman of the Department of Physiotherapy & Rehabilitation at Jashore University of Science & Technology will act as the coordinator of the trial monitoring team, assisted by two other volunteers from the Master of Physiotherapy program who are not involved in any aspect of this trial.

### Safety measures and managing adverse effects

All procedures will be carried out under the supervision of the trial monitoring team and a qualified physician with expertise in managing diabetic peripheral neuropathy (DPSN) in order to ensure the participants’ safety.

1. Manageable adverse events: Temporary increase in pain or tingling sensation and mild muscle soreness or fatigue**.**
2. Serious adverse events: Considered fatal are severe nerve injury leading to permanent loss of sensation or motor function, diabetic foot ulceration, or any intervention-related complications resulting in infection or vascular compromise**.**

In addition to taking appropriate clinical measures, such as changing or stopping the intervention if needed, any adverse incidents will be documented and submitted to the ethics committee.

### Discussion

Diabetic peripheral neuropathy (DPSN) is a common complication of diabetes that affects nerve function, causing pain, numbness, and mobility issues. This study will evaluate the effectiveness of neurodynamic mobilization combined with foot exercises in improving nerve function, reducing pain, and enhancing quality of life in individuals with DPSN. It is anticipated that the findings of this study will support the idea that neurodynamic mobilization can be an effective non-pharmacological treatment for DPSN. Participants allocated to the neurodynamic mobilization group are expected to show greater improvements in pain, nerve function, and functional mobility compared to those in the control group. These expected outcomes will be consistent with existing literature, which suggests that nerve mobilization techniques may enhance intraneural blood flow, decrease mechanical stiffness, and promote optimal nerve gliding, thereby contributing to symptom relief. Furthermore, this study aims to generate additional evidence on the clinical applicability of neurodynamic techniques in the conservative management of DPSN. The findings of this trial will also help to clarify the role of such interventions within rehabilitation practice, particularly in settings with limited access to pharmacological management options [4–8].

One of the key findings of this study will be the improvement in the Michigan Diabetic Neuropathy Score (MDNS) among participants in the experimental group. The reduction in MDNS scores will suggest that neurodynamic mobilization positively impacts nerve function and reduces sensory disturbances. On the other hand, improvements in the Visual Analog Scale (VAS) scores will indicate that participants experienced less pain after receiving the intervention. This will be important because chronic pain is a major concern for individuals with DPN, affecting their daily activities and quality of life [55,56]. Another significant outcome of this study will be the improvement in Neuropathy-Specific Quality of Life (Neuro-QoL) scores**.** Participants in the experimental group reported better physical function, reduced pain-related limitations, and improved overall well-being [45–50]. This suggests that neurodynamic mobilization, along with foot exercises, may enhance daily functioning and reduce the risk of further complications, such as falls and foot ulcers [11,12]. Possible explanations for these improvements are that neurodynamic mobilization promotes nerve gliding and reduces mechanical stress on nerves, which will help restore normal nerve function. The techniques used in this study will target key nerves affected in DPSN, including the sciatic, tibial, sural, and common peroneal nerves. By enhancing nerve mobility, the intervention may have improved sensory and motor function in the lower limbs [54,57,58]. The inclusion of foot exercises in both groups will have also contributed to positive outcomes. These exercises will help improve sensory input, circulation, and muscle strength, which are essential for maintaining foot health in individuals with DPSN [53,54,57,58]. The control group will also show some improvement, likely due to the benefits of foot exercises and Transcutaneous Electrical Nerve Stimulation (TENS) therapy, which is known to reduce pain and improve circulation. However, the experimental group will show greater improvements, indicating that neurodynamic mobilization provides additional benefits [41–45].

Despite these promising findings, this study will have some limitations**.** The sample size may be relatively small, and the study will be conducted at a single center, which may limit the generalizability of the results. Additionally, the long-term effects of neurodynamic mobilization were not assessed beyond the 12-week follow-up period. Future studies should include larger sample sizes, multiple study centers, and longer follow-up periods to determine whether the benefits of neurodynamic mobilization are sustained over time [52–54,57,58].

### Conclusion

This study will provide evidence that neurodynamic mobilization, combined with foot exercises, can significantly improve nerve function, reduce pain, and enhance the quality of life in individuals with DPSN. It offers a promising non-pharmacological treatment approach for managing diabetic neuropathy symptoms. Further research is needed to confirm these findings and to explore the long-term effects of neurodynamic mobilization in individuals with diabetes.

## Supporting information

S1 File SPIRIT checklist. (DOCX)

S2 File Study appendix. (PDF)
