# Supplementary material for: Study protocol for a double-blinded randomized clinical trial evaluating effectiveness of neurodynamic approach in lower limb for diabetic peripheral sensory neuropathy
Source: PLoS One. 2026 May 12;21(5):e0348347. doi: 10.1371/journal.pone.0348347 (PMC13166923; doi:10.1371/journal.pone.0348347)
Supplement: S2 File — (PDF) [file pone.0348347.s002.pdf]

EC Approved Study Protocol:

Title: A double-blinded Randomized clinical trial Effectiveness of Neurodynamic approach in lower limb for Diabetic Peripheral Sensory Neuropathy

SL No	Documents	Version	*No of copies*
1	Ethical statement checklist	1	1
2	Research methodology	1	1
3	Participant Information Sheet (PIS)	1	1
4	Informed consent form	1	1
5	Questionnaire	1	1
6	Intervention tracking sheet/ checklist (for Experimental study)	1	1
7	Randomization sequencing (for Experimental study)	1	1
8	Adverse effect tracking and management form (for Experimental study)	1	1
9	Time Frame: (Gantt chart)	1	1
10	Budget	1	1

1. Annexure: A

Put Tick sign (√) appropriate answers against each of the following statement (If not Applicable, Please write NA)

1. Source of Population:

a) Patients ☑ Yes ☐ No
b) Healthy Subjects ☐Yes ☑ No
c) Minors or person under guardianship ☐Yes ☑ No

2. Does the study involve:

a) Physical risks to the Subjects ☐Yes ☑ No
b) Social Risks ☐Yes ☑ No
c) Psychological risks ☐Yes ☑ No
d) Discomfort to subjects ☐Yes ☑ No
e) Invasion of the body ☐Yes ☑ No
f) Invasion of Privacy ☐Yes ☑ No
g) Disclosure of information damaging to subject or others ☐Yes ☑ No

3. Does the study involve :

a) Use of records :- (Hospital, Medical, Death, Birth or other) ☑Yes ☐ No
b) Use of foetal tissues or abortus ☐ Yes ☑ No
c) Use of organs or body fluids ☐ Yes ☑ No

4. Are subjects clearly informed about:

a) Nature and purposes of study ☑ Yes ☐ No
b) Procedures to be followed including alternative used ☐ Yes ☑ No
c) Physical risks ☐ Yes ☑ No
d) Private questions ☐ Yes ☑ No
e) Mental risks ☐ Yes ☑ No
f) Benefits to be derived ☐Yes ☑ No
g) Right to refuse to participate or to withdraw from study ☑ Yes ☐ No
h) Confidential handling of data ☑ Yes ☐ No
i) Compensations: (where there are risks or loss of working time or privacy is involved in any particular procedure) ☐ Yes ☑ No

5. Signed consent form will be obtained:

a) From Subjects(If adult) ☑ Yes ☐ No
b) From parent or guardian (if subjects are minor) ☐ Yes ☑ No

6. Will precautions be taken to protect anonymity of subjects? ☑ Yes ☐ No

2. Annexure: B

Methodology

This study will be a patient and assessor-blinded randomized clinical trial (RCT) conducted at Nurul Islam Diabetic Center, Jashore, Bangladesh. Ninety (90) participants diagnosed with diabetic peripheral sensory neuropathy (DPSN) will be randomly allocated (1:1) into the Experimental Group (Neurodynamic Mobilization) and Control Group (Sham Neurodynamic Mobilization) using block randomization. Both groups will receive common foot exercises and transcutaneous electrical nerve stimulation (TENS). The intervention will be administered three week ly for four weeks, with follow-ups at eight weeks post-treatment.

Primary outcomes include the Michigan Diabetic Neuropathy Score (MDNS), while secondary outcomes comprise the Visual Analog Scale (VAS) for pain and the Neuropathy-Specific Quality of Life Questionnaire (Neuro-QoL). We will use the Kolmogorov-Smirnov normality test to investigate the sample size's normal distribution. The proper statistical tests, including mean, standard deviation (SD), and level of significance (LOS) maintained at 0.05 with a 95% confidence interval (CI), will be used to meet the study's objectives. Repeated measures independent t-test will be used to analyze the among-group data analysis if it is normally distributed; if not, the Friedman test will be applied. Post-hoc tests will be performed by Mann U Whitney test or independent t-test based on the nature of data. We will use an intention to treat analysis and Bonferroni correction will be made for the post-hoc tests.

3. Annexure: C

Department of Physiotherapy & Rehabilitation

Jashore University of Science & Technology

Free Physiotherapy Treatment for Diabetic Neuropathy Patients
Diabetic neuropathy patients will receive free physiotherapy treatment for 4 weeks.

Diabetic Neuropathy (120 Patients)
1) Age: 40-70 years, both male and female<br>2) Type 1 or Type 2 Diabetes Mellitus<br>3) Symptoms in the back of the foot or entire foot:<br>a) Tingling, numbness<br>b) Burning sensation, pain<br>c) Inability to sense hot or cold<br>d) Weakness, lack of strength in the foot, muscle atrophy<br>4) Diagnosed with diabetic neuropathy by a physician

Treatment Centers

Department of Physiotherapy and Rehabilitation<br>Dr. M R Khan Medical Center ;<br>Jessore University of Science and Technology	Nurul Islam Diabetes Center<br><br>West of Fire Service Office, Neel Ratan Dhar Road, Jessore

What Will Patients Receive?	Why Are We Providing Free Treatment?
1. Complete diagnosis and treatment free of cost<br>2. Post-treatment consultation and advice<br>3. Confidentiality of all medical information	1. The university is committed to serving the community<br>2. To explore more effective treatment methods<br>3. As part of education and research activities

Contact for Free Treatment

Ambika Kormoker<br>4th Professional Year Student, JUST<br>01779448587,<br>ambikakormokar56@gmail.com	Dr. Kazi Md. Emran Hossain*<br>Lecturer, Department of Physiotherapy and Rehabilitation<br>01735661492; kma.hossain@just.edu.bd

4. Annexure: D

Informed consent Form

If you agree to participate in this study, please confirm by putting your signature/thumbprint.

Thank you for your cordial cooperation.

(Note to investigator: Please note that the items below can vary from protocol to protocol)

Do you give permission for the interview to be audio recorded? *Please note that at any point during the interview, you can ask the interviewer to turn off the recording device.*	☑Yes	No
Do you give permission to share your findings?	☑Yes	No
Do you give permission to take photos?	☑Yes	No
Do you give permission to share the photos?	☑Yes	No
Do you give permission to receive specimen (blood/ tissue)?	☑Yes	No
Do you give permission for intervention?	☑Yes	No
(If you need to contact the/collect data from the respondent again, need to state here and ask permission…)		
Do you have any question?	Yes	No

State the question

**Interviewer name:**	**Respondent's name:**
**Interviewer signature:**	**Respondent's signature/thumbprint:**
**Date:**	**Date:**

5. Questionnaire:

INFORMED CONSENT

Research Name: "A double-blinded Randomized clinical trial Effectiveness of Neurodynamic approach in lower limb for Diabetic Peripheral Sensory Neuropathy"

Assalamualaikum/Namasker,

My name is Ambika Kormoker, a 5th year student of studing Bachelor of physiotherapy (BPT) at Physiotherapy and Rehabilitation Department, Jashore University of Science and Technology. I am conducting this study under guidance of Dr. Kazi Md. Amran Hossain (PT), lecturer of the Physiotherapy and Rehabilitation Department, Jashore University of Science and Technology. My research topic is **"A double-blinded Randomized clinical trial Effectiveness of Neurodynamic approach in lower limb for Diabetic Peripheral Sensory Neuropathy".**

I would like to know about some personal and other related information about neurological problem among the diabetic patients. This will take approximately 15-20 minutes. If you feel any type of discomfort or disturbances including physical, emotional, and social risks you can share them with me, or if you don't want to continue, I will stop the interview. Your participation is completely voluntary. It is your choice whether to participate or not and you will get no payment for taking part in this study. You can leave the questionnaire anytime during this question period

I would also like to assure you that all data will be kept confidential and will not be used for any other purpose. During publication, your personal information will not be disclosed. You have the right to withdraw your participation at any time in your study without any negative consequences in your treatment. You also have the right to skip any question that you do not want to answer during the interview. So, I am requesting you be a part of this research.This study has been registered to the Clinical Trials Registry India (CTRI).

If you have something know about the research , you may contact with me(Ambika-01779448587) and/or my research supervisor Dr. Kazi Md. Amran Hossain (phone: 01735661492,mail: kma.hossain@just.edu.bd) .So, may I have your consent to proceed with the interview or work?

Yes ☐   No ☐

Name of Participant: Address:

Mobile number: Code no:

Signature of the Participant: ______________________________

Witness's signature: ____________________________________

Data collector's signature:___________________Date of Data Collection: ______________

Research Title: "A double-blinded Randomized clinical trial Effectiveness of Neurodynamic approach in lower limb for Diabetic Peripheral Sensory Neuropathy"

Questionnaire

Section 1: Socio-Demographic Information

1	Patient Code no:	2	Age: ___________________Years
3	Gender: 1. Female<br>2. Male	4	Living area:<br>1. Urban<br>2. Semi urban<br>3. Rural
5	Educational status:<br>1. Illiterate<br>2. Primary<br>3. Secondary school certificate<br>4. Higher secondary certificate<br>5. Graduate<br>6. Masters or above<br>Others:	6	Occupation:<br>1. Government Service holder<br>2. Private Service holder<br>3. Housewife<br>4. Day labor<br>5. Businessman<br>6. Garments worker<br>7. Unemployed<br>8. Others (specify):
7	How many hours do you work in a day?	8	BMI :<br>Height:<br>Weight:
9	Diabetic Type:<br>1. Type I<br>2. Type II	10	What is the duration of diabetes suffering?
11	What type of treatment you are taking for diabetes?<br>1. Only food control<br>2. Food maintenance and medication<br>3. Only medication<br>4. Physical exercise<br>5. Insulin<br>6. Nothing<br>7. Combination of 1,2,4	12	Do you Smoke?<br>1. Daily<br>2. Occasionally<br>3. Never<br>4. Stopped
13	Do you exercise?<br>1. Yes<br>2. No	14	Duration of exercise per day-

<u>**Before Treatment (Pretest)**</u>

Section 2: Symptom & paraesthesia related Information

1.(a) Location of symptom:

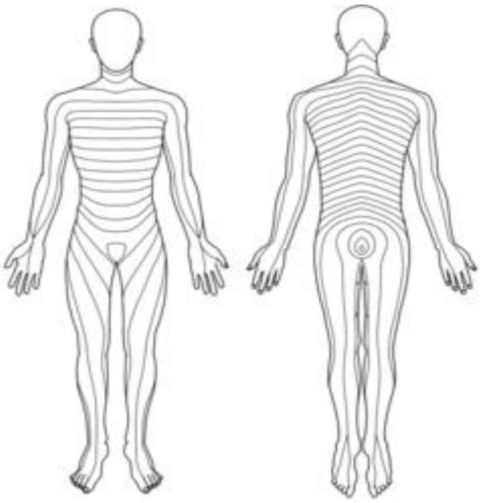

VAS Score: (10 cm)

0 ———————————————————————— 10cm

No pain **Extreme pain**

Section 3:THE MICHIGAN DIABETIC NEUROPATHY SCORE (MDNS)

**MDNS items**			
Sensory impairment:	Right	Vibration at big toe	☐ 0 = Normal ☐ 1=Decrease ☐ = Absent
		10-g filament	☐ 0 = Normal ☐ 1=Decrease ☐ = Absent
		Pin prick on dorsum of the great toe	☐ 0= Painful ☐ 2=Not painful
	Left	Vibration at big toe	☐ 0 = Normal ☐ 1=Decrease ☐ = Absent
		10-g filament	☐ 0 = Normal ☐ 1=Decrease ☐ = Absent
		Pin prick on dorsu m of the great toe	☐ 0= Painful ☐ 2=Not painful
Muscle strength testing	Right	Finger spread	☐ 0 = Normal ☐ 1 =Mild to moderate<br>☐ 2 =Sever ☐ 3= Absent
		Great toe extension	☐ 0 = Normal ☐ 1 =Mild to moderate<br>☐ 2 =Sever ☐ 3= Absent

		Ankle dorsiflexion	☐ 0 = Normal ☐ 1 =Mild to moderate<br>☐ 2 =Sever ☐ 3= Absent
	Left	Finger spread	☐ 0 = Normal ☐ 1 =Mild to moderate<br>☐ 2 =Sever ☐ 3= Absent
		Great toe extension	☐ 0 = Normal ☐ 1 =Mild to moderate<br>☐ 2 =Sever ☐ 3= Absent
		Ankle dorsiflexion	☐ 0 = Normal ☐ 1 =Mild to moderate<br>☐ 2 =Sever ☐ 3= Absent
**Reflexs**	Right	Biceps brachii	☐ 0 = Present ☐ 1 =Present with reinforcement<br>☐ 2 =Absent
		Triceps brachii	☐ 0 = Present ☐ 1 =Present with reinforcement<br>☐ 2 =Absent
		Quadriceps femoris	☐ 0 = Present ☐ 1 =Present with reinforcement<br>☐ 2 =Absent
		Achilles	☐ 0 = Present ☐ 1 =Present with reinforcement<br>☐ 2 =Absent
	Left	Biceps brachii	☐ 0 = Present ☐ 1 =Present with reinforcement<br>☐ 2 =Absent
		Triceps brachii	☐ 0 = Present ☐ 1 =Present with reinforcement<br>☐ 2 =Absent
		Quadriceps femor is	☐ 0 = Present ☐ 1 =Present with reinforcement<br>☐ 2 =Absent
		Achilles	☐ 0 = Present ☐ 1 =Present with reinforcement<br>☐ 2 =Absent

Total ___________________________/46 points

Section 3: Neuropathy Specific Quality of Life Questionnaire (Neuro QoL)

**NeuroQol items**		**After treatment**
**1**	Burning in your legs or feet.	☐1=Never ☐ 2=Rarely ☐ 3=Sometimes<br>☐ 4=Often ☐ 5=Always
**2**	Excessive heat or cold in your legs or feet	☐ 1=Never ☐ 2=Rarely ☐ 3=Sometimes<br>☐ 4=Often ☐ 5=Always
**3**	Pins and pricks in your legs or feet	☐ 1=Never ☐ 2=Rarely ☐ =Sometimes<br>☐ 4=Often ☐ 5=Always
**4**	Acute or piercing pain in your legs or feet.	☐ 1=Never ☐ 2=Rarely ☐ 3=Sometimes<br>☐ 4=Often ☐ 5=Always
**5**	Pounding in your legs or feet	☐1=Never ☐ 2=Rarely ☐ 3=Sometimes<br>☐ 4=Often ☐ 5=Always
**6**	Feelings in your legs or feet that make them jump.	☐ 1=Never ☐ 2=Rarely ☐ 3=Sometimes

		☐ 4=Often ☐ 5=Always
**7**	Skin irritation caused by something in contact with your feet, sheets or socks, for example	☐1=Never ☐ 2=Rarely ☐ 3=Sometimes<br>☐ 4=Often ☐ 5=Always
**8**	Numbness in your feet	☐ 1=Never ☐ 2=Rarely ☐ 3=Sometimes<br>☐ 4=Often ☐ 5=Always
**9**	Inability to feel the difference between hot and cold with your feet	☐1=Never ☐ 2=Rarely ☐ 3=Sometimes<br>☐ 4=Often ☐ 5=Always
**10**	Inability to feel objects with your feet	☐ 1=Never ☐ 2=Rarely ☐ 3=Sometimes<br>☐ 4=Often ☐ 5=Always
**11**	Weakness in your hands	☐1=Never ☐ 2=Rarely ☐ 3=Sometimes<br>☐ 4=Often ☐ 5=Always
**12**	problems or instability when walking	☐ 1=Never ☐ 2=Rarely ☐ 3=Sometimes<br>☐ 4=Often ☐ 5=Always
**13**	Balance problems or instability when standing	☐1=Never ☐ 2=Rarely ☐ 3=Sometimes<br>☐ 4=Often ☐ 5=Always
**As a result of foot problems:**		
**14**	Your self-confidence has been affected	☐ 1=Not at all ☐ 2=A little bit ☐ 3=Somewhat<br>☐ 4=Quite a bit ☐ 5=Very much
**15**	You feel older than your years	☐ 1=Not at all ☐ 2=A little bit ☐ 3=Somewhat<br>☐ 4=Quite a bit ☐ 5=Very much
**16**	Your life is a struggle	☐ 1=Not at all ☐ 2=A little bit ☐ 3=Somewhat<br>☐ 4=Quite a bit ☐ 5=Very much
**17**	You feel frustrated	☐ 1=Not at all ☐ 2=A little bit ☐ 3=Somewhat<br>☐ 4=Quite a bit ☐ 5=Very much
**18**	You feel embarrassed	☐ 1=Not at all ☐ 2=A little bit ☐ 3=Somewhat<br>☐ 4=Quite a bit ☐ 5=Very much
**19**	You feel depressed	☐ 1=Not at all ☐ 2=A little bit ☐ 3=Somewhat<br>☐ 4=Quite a bit ☐ 5=Very much
**20**	Foot problems interfere with close relationships	☐ 1=Never ☐ 2=Rarely ☐ 3=Sometimes<br>☐ 4=Often ☐ 5=Always
**As a result of foot problems:**		
**21**	You feel more physically dependent	☐ 1=Never ☐ 2=Rarely ☐ 3=Sometimes<br>☐ 4=Often ☐ 5=Always
**22**	You feel more emotionally dependent	☐1=Never ☐ 2=Rarely ☐ 3=Sometimes<br>☐ 4=Often ☐ 5=Always
**23**	Your role in family changed	☐ 1=Never ☐ 2=Rarely ☐ 3=Sometimes<br>☐ 4=Often ☐ 5=Always
**24**	You are treated differently	☐1=Never ☐ 2=Rarely ☐ 3=Sometimes<br>☐ 4=Often ☐ 5=Always
**Foot problems interfere with:**		

**25**	Ability to perform paid work	☐ 1=Never ☐ 2=Rarely ☐ 3=Sometimes ☐ 4=Often ☐ 5=Always
**26**	Ability to perform daily tasks	☐1=Never ☐ 2=Rarely ☐ 3=Sometimes ☐ 4=Often ☐ 5=Always
**27**	Ability to take part in leisure activities	☐ 1=Never ☐ 2=Rarely ☐ 3=Sometimes ☐ 4=Often ☐ 5=Always

After Treatment (Posttest)

Section 2: Symptoms & paraesthesia

Related Information-

1. (a) Location of symptom:

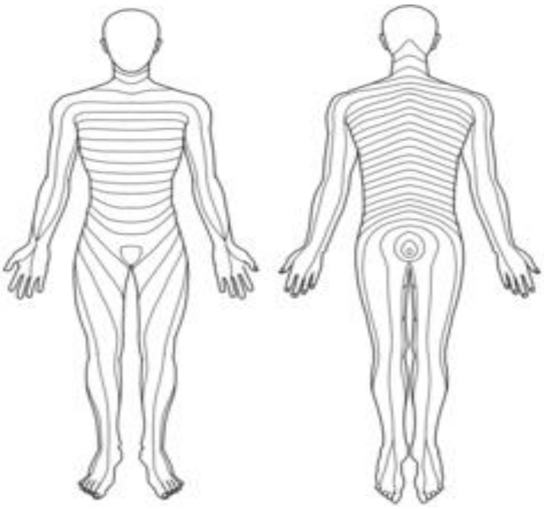

VAS Score: (10 cm)

(0) **No pain** **Extreme pain(** 10cm)

Section 3: THE MICHIGAN DIABETIC NEUROPATHY SCORE (MDNS)

**MDNS items**			
Sensory impairment:	Right	Vibration at big toe	☐ 0 = Normal ☐ 1=Decrease ☐ = Absent
		10-g filament	☐ 0 = Normal ☐ 1=Decrease ☐ = Absent
		Pin prick on dorsum of the	☐ 0= Painful ☐ 2=Not painful

		great toe	
	Left	Vibration at big toe	☐ 0 = Normal ☐ 1=Decrease ☐ = Absent
		10-g filament	☐ 0 = Normal ☐ 1=Decrease ☐ = Absent
		Pin prick on dorsum of the great toe	☐ 0= Painful ☐ 2=Not painful
Muscle strength testing	Right	Finger spread	☐ 0 = Normal ☐ 1 =Mild to moderate<br>☐ 2 =Sever ☐ 3= Absent
		Great toe extension	☐ 0 = Normal ☐ 1 =Mild to moderate<br>☐ 2 =Sever ☐ 3= Absent
		Ankle dorsiflexion	☐ 0 = Normal ☐ 1 =Mild to moderate<br>☐ 2 =Sever ☐ 3= Absent
	Left	Finger spread	☐ 0 = Normal ☐ 1 =Mild to moderate<br>☐ 2 =Sever ☐ 3= Absent
		Great toe extension	☐ 0 = Normal ☐ 1 =Mild to moderate<br>☐ 2 =Sever ☐ 3= Absent
		Ankle dorsiflexion	☐ 0 = Normal ☐ 1 =Mild to moderate<br>☐ 2 =Sever ☐ 3= Absent
Reflexs	Right	Biceps brachii	☐ 0 = Present ☐ 1 =Present with reinforcement<br>☐ 2 =Absent
		Triceps brachii	☐ 0 = Present ☐ 1 =Present with reinforcement<br>☐ 2 =Absent
		Quadriceps femoris	☐ 0 = Present ☐ 1 =Present with reinforcement<br>☐ 2 =Absent
		Achilles	☐ 0 = Present ☐ 1 =Present with reinforcement<br>☐ 2 =Absent
	Left	Biceps brachii	☐ 0 = Present ☐ 1 =Present with reinforcement<br>☐ 2 =Absent
		Triceps brachii	☐ 0 = Present ☐ 1 =Present with reinforcement<br>☐ 2 =Absent
		Quadriceps femoris	☐ 0 = Present ☐ 1 =Present with reinforcement<br>☐ 2 =Absent
		Achilles	☐ 0 = Present ☐ 1 =Present with reinforcement<br>☐ 2 =Absent

Total ____________________________/46 points

Section 3: Neuropathy Specific Quality of Life Questionnaire (Neuro QoL)

NeuroQol items		After treatment
**1**	Burning in your legs or feet.	☐1=Never ☐ 2=Rarely ☐ 3=Sometimes ☐ 4=Often ☐ 5=Always
**2**	Excessive heat or cold in your legs or feet	☐ 1=Never ☐ 2=Rarely ☐ 3=Sometimes ☐ 4=Often ☐ 5=Always
**3**	Pins and pricks in your legs or feet	☐ 1=Never ☐ 2=Rarely ☐ =Sometimes ☐ 4=Often ☐ 5=Always
**4**	Acute or piercing pain in your legs or feet.	☐ 1=Never ☐ 2=Rarely ☐ 3=Sometimes ☐ 4=Often ☐ 5=Always
**5**	Pounding in your legs or feet	☐1=Never ☐ 2=Rarely ☐ 3=Sometimes ☐ 4=Often ☐ 5=Always
**6**	Feelings in your legs or feet that make them jump.	☐ 1=Never ☐ 2=Rarely ☐ 3=Sometimes ☐ 4=Often ☐ 5=Always
**7**	Skin irritation caused by something in contact with your feet, sheets or socks, for example	☐1=Never ☐ 2=Rarely ☐ 3=Sometimes ☐ 4=Often ☐ 5=Always
**8**	Numbness in your feet	☐ 1=Never ☐ 2=Rarely ☐ 3=Sometimes ☐ 4=Often ☐ 5=Always
**9**	Inability to feel the difference between hot and cold with your feet	☐1=Never ☐ 2=Rarely ☐ 3=Sometimes ☐ 4=Often ☐ 5=Always
**10**	Inability to feel objects with your feet	☐ 1=Never ☐ 2=Rarely ☐ 3=Sometimes ☐ 4=Often ☐ 5=Always
**11**	Weakness in your hands	☐1=Never ☐ 2=Rarely ☐ 3=Sometimes ☐ 4=Often ☐ 5=Always
**12**	problems or instability when walking	☐ 1=Never ☐ 2=Rarely ☐ 3=Sometimes ☐ 4=Often ☐ 5=Always
**13**	Balance problems or instability when standing	☐1=Never ☐ 2=Rarely ☐ 3=Sometimes ☐ 4=Often ☐ 5=Always
**As a result of foot problems:**		
**14**	Your self-confidence has been affected	☐ 1=Not at all ☐ 2=A little bit ☐ 3=Somewhat ☐ 4=Quite a bit ☐ 5=Very much
**15**	You feel older than your years	☐ 1=Not at all ☐ 2=A little bit ☐ 3=Somewhat ☐ 4=Quite a bit ☐ 5=Very much
**16**	Your life is a struggle	☐ 1=Not at all ☐ 2=A little bit ☐ 3=Somewhat ☐ 4=Quite a bit ☐ 5=Very much
**17**	You feel frustrated	☐ 1=Not at all ☐ 2=A little bit ☐ 3=Somewhat ☐ 4=Quite a bit ☐ 5=Very much
**18**	You feel embarrassed	☐ 1=Not at all ☐ 2=A little bit ☐ 3=Somewhat ☐ 4=Quite a bit ☐ 5=Very much
**19**	You feel depressed	☐ 1=Not at all ☐ 2=A little bit ☐ 3=Somewhat

		☐ 4=Quite a bit ☐ 5=Very much
**20**	Foot problems interfere with close relationships	☐ 1=Never ☐ 2=Rarely ☐ 3=Sometimes ☐ 4=Often ☐ 5=Always
**As a result of foot problems:**		
**21**	You feel more physically dependent	☐ 1=Never ☐ 2=Rarely ☐ 3=Sometimes ☐ 4=Often ☐ 5=Always
**22**	You feel more emotionally dependent	☐1=Never ☐ 2=Rarely ☐ 3=Sometimes ☐ 4=Often ☐ 5=Always
**23**	Your role in family changed	☐ 1=Never ☐ 2=Rarely ☐ 3=Sometimes ☐ 4=Often ☐ 5=Always
**24**	You are treated differently	☐1=Never ☐ 2=Rarely ☐ 3=Sometimes ☐ 4=Often ☐ 5=Always
**Foot problems interfere with:**		
**25**	Ability to perform paid work	☐ 1=Never ☐ 2=Rarely ☐ 3=Sometimes ☐ 4=Often ☐ 5=Always
**26**	Ability to perform daily tasks	☐1=Never ☐ 2=Rarely ☐ 3=Sometimes ☐ 4=Often ☐ 5=Always
**27**	Ability to take part in leisure activities	☐ 1=Never ☐ 2=Rarely ☐ 3=Sometimes ☐ 4=Often ☐ 5=Always

6. Intervention tracking sheet/checklis (Put tick √)

Patient ID	Group (E/C)	Pretest	Intervention	Posttest
P1				
P2				
P3				
P4				
P5				
P6				
P7				
P8				
P9				
P10				
P11				
P12				
P13				
P14				
P15				
P16				
P17				
P18				
P19				
PP20				
P21				
P22				
P23				
P24				
P25				
P26				
P27				
P28				
P29				
P30				
P31				
P32				
P33				
P34				
P35				
P36				
P37				
P38				

P39				
P40				
P41				
P42				
P43				
P44				
P45				
P46				
P47				
P48				
P49				
P50				
P51				
P52				
P53				
P54				
P55				
P56				
P57				
P58				
P59				
P60				
P61				
P62				
P63				
P64				
P65				
P66				
P67				
P68				
P69				
P70				
P71				
P72				
P73				
P74				
P75				
P76				
P77				
P78				
P79				
P80				

7. Randomization sequencing:

Patient ID	RAND	Group
P19	0.016341	Control group
P75	0.029208	Experimental group
P24	0.116292	Control group
P36	0.119401	Experimental group
P2	0.124213	Control group
P20	0.129289	Experimental group
P66	0.142846	Control group
P11	0.149819	Experimental group
P52	0.153534	Control group
P13	0.15966	Experimental group
P48	0.161842	Control group
P37	0.182973	Experimental group
P40	0.19792	Control group
P21	0.210004	Experimental group
P44	0.216444	Control group
P41	0.224634	Experimental group
P17	0.231808	Control group
P43	0.232833	Experimental group
P69	0.263246	Control group
P6	0.274508	Experimental group
P60	0.28407	Control group
P74	0.298362	Experimental group
P47	0.326748	Control group
P29	0.341563	Experimental group
P33	0.378403	Control group
P16	0.396658	Experimental group
P56	0.397543	Control group
P5	0.398945	Experimental group
P25	0.402663	Control group
P32	0.414455	Experimental group
P68	0.434967	Control group
P3	0.464241	Experimental group
P27	0.466463	Control group
P71	0.483572	Experimental group
P49	0.521414	Control group
P45	0.525382	Experimental group
P67	0.532303	Control group
P79	0.537093	Experimental group
P70	0.544932	Control group
P53	0.559824	Experimental group
P26	0.610127	Control group
P72	0.614551	Experimental group
P38	0.619329	Control group

P18	0.622271	Experimental group
P50	0.626752	Control group
P8	0.628661	Experimental group
P39	0.653032	Control group
P4	0.656519	Experimental group
P76	0.659769	Control group
P59	0.665966	Experimental group
P78	0.669214	Control group
P28	0.674386	Experimental group
P7	0.674881	Control group
P58	0.681739	Experimental group
P10	0.704246	Control group
P54	0.710106	Experimental group
P35	0.719633	Control group
P23	0.728408	Experimental group
P62	0.73141	Control group
P15	0.734016	Experimental group
P80	0.771242	Control group
P55	0.804461	Experimental group
P65	0.814515	Control group
P9	0.835712	Experimental group
P51	0.841259	Control group
P57	0.841369	Experimental group
P77	0.850954	Control group
P30	0.851963	Experimental group
P61	0.855677	Control group
P34	0.858116	Experimental group
P14	0.884058	Control group
P12	0.897036	Experimental group
P64	0.920832	Control group
P63	0.938639	Experimental group
P22	0.95733	Control group
P1	0.961082	Experimental group
P46	0.97044	Control group
P31	0.979044	Experimental group
P73	0.986034	Control group
P42	0.987676	Experimental group

8. Adverse effect checklist :(Put tick √ and write comment if any adverse effect notice and put cross X mark if no adverse effect notice)

1. **Myalgia (Muscle aches and Pain)**

Patient ID	Group	Pretest	Intervention	Posttest
P1				
P2				
P3				
P4				
P5				
P6				
P7				
P8				
P9				
P10				
P11				
P12				
P13				
P14				
P15				
P16				
P17				
P18				
P19				
PP20				
P21				
P22				
P23				
P24				
P25				
P26				
P27				
P28				
P29				
P30				
P31				
P32				
P33				
P34				
P35				
P36				
P37				
P38				

P39				
P40				
P41				
P42				
P43				
P44				
P45				
P46				
P47				
P48				
P49				
P50				
P51				
P52				
P53				
P54				
P55				
P56				
P57				
P58				
P59				
P60				
P61				
P62				
P63				
P64				
P65				
P66				
P67				
P68				
P69				
P70				
P71				
P72				
P73				
P74				
P75				
P76				
P77				
P78				
P79				
P80				

9. Time Frame: (Gantt chart)

Activities	Mon/ Yr	Mon/ Yr	Mon/ Yr	Mon/ Yr	Mon/ Yr	Mon/ Yr	Mon/ Yr	Mon/ Yr	Mon/ Yr	Mon/ Yr
**Designing the Study**	Jan 2025									
**Review of Literature**	Jan 2025	Feb 2025								
**Development & approval of proposal**	Jan 2025									
**Development of Data Collection Tools**		Feb 2025								
**Pre testing Questionnaire**			Mar 2025							
**Data Collection, Entry & Analysis**				April 2025	May 2025	June 2025	July 2025	Aug 2025		
**Report Writing**								Aug 2025	Sept. 2025	
**Submission & Approval**										Oct 2025
**Submission for Publication**										Nov 2025

10. Budget

Questionnaire printing	8,000tk
Measuring tools	10,000tk
Data Collection	6,000tk
Transport Transport	10,000tk
Physiotherapist(male, female) remuneration	60,000tk
Research assistance(male, female) remuneration	20,000tk
Miscellaneous	10,000tk
Total	124,000tk
5% increment	6,200tk
Grand Total10,342tk	130,200tk
